# *Dirofilaria immitis* in a child from the Russian Federation

**DOI:** 10.1051/parasite/2016037

**Published:** 2016-09-07

**Authors:** Nelli Ignatievna Tumolskaya, Edoardo Pozio, Vera Mikhaylovna Rakova, Valentina Georgievna Supriaga, Vladimir Petrovich Sergiev, Evgeny Nikolaevich Morozov, Lola Farmonovna Morozova, Giovanni Rezza, Serguei Kirillovich Litvinov

**Affiliations:** 1 Martsinovsky Research Institute on Medical Parasitology and Tropical Medicine, Sechenov First Moscow State Medical University M. Pirigovskaya str. 20 119495 Moscow Russian Federation; 2 Istituto Superiore di Sanità viale Regina Elena 299 00161 Rome Italy; 3 Tropical Medicine and Parasitic Diseases, Sechenov First Moscow State Medical University M. Pirogovskaya str. 20 119495 Moscow Russian Federation

**Keywords:** *Dirofilaria immitis*, Dirofilariosis, Morphology, Molecular identification, Zoonosis, Epidemiology

## Abstract

An immature female worm, *Dirofilaria immitis*, was isolated from the scrotum of a 14-month-old child. This is the first identification of human dirofilariosis caused by *D. immitis* in a relatively Northern region (Moscow) of the Russian Federation. The parasite was diagnosed by means of morphological examination of the worm, confirmed by PCR assay. This case raises questions about the range of the *D. immitis* distribution among humans in Russia. To better understand the geographical distribution of dirofilarioses, detailed clinical and epidemiological information should be collected from human cases with appropriate laboratory confirmation.

## Abstract (fr)

Un ver femelle immature, *Dirofilaria immitis*, a été isolé du scrotum d’un enfant âgé de 14 mois. Ceci est la première identification de dirofilariose humaine causée par *D. immitis* dans une région relativement nordique (Moscou) de la Fédération de Russie. Le parasite a été diagnostiqué par un examen morphologique du ver, confirmé par PCR. Ce cas soulève des questions sur la distribution de *D. immitis* chez l’homme en Russie. Pour mieux comprendre la répartition géographique des dirofilarioses, des informations cliniques et épidémiologiques détaillées doivent être recueillies à partir des cas humains, avec confirmation appropriée en laboratoire.

## Abstract (ru)

Heпoлoвoзpeлaя жeнcкaя ocoбь чepвя *Dirofilaria immitis* былa изoлиpoвaнa из cкpoтyмa 14 - мecячнoгo peбeнкa. Этo пepвый cлyчaй выявлeния диpoфиляpиoзa чeлoвeкa, вызвaннoгo *D. immitis* в cpaвнитeльнo ceвepнoм peгиoнe (Mocквa) Poccийcкoй Фeдepaции. Пapaзит был диaгнocтиpoвaн мopфoлoгичecки и мeтoдoм пoлимepaзнoй цeпнoй peaкции. Этoт cлyчaй пoднимaeт вoпpoc oтнocитeльнo ypoвня pacпpocтpaнeния *D. immitis* cpeди нaceлeния Poccии. Для лyчшeгo пoнимaния гeoгpaфичecкoгo pacпpocтpaнeния диpoфиляpиoзa тpeбyютcя бoлee дeтaльный клиничecкий и эпидeмиoлoгичecкий aнaлиз кaждoгo cлyчaя диpoфиляpиoзa чeлoвeкa, пoдтвepждeннoгo лaбopaтopнo.

## Introduction

In the Russia Federation, dirofilariosis is commonly identified in domestic and wild carnivores [21]. *Dirofilaria repens* has been documented in dogs and cats, whereas *Dirofilaria immitis* has been reported in dogs of the South and the Far East of Russia [2]. *Dirofilaria ursi* has been reported in brown bears (*Ursus arctos*) of the Primorskiy kray, Sakhalin, Yakutia, and of the Vologda regions [2, 21]. Human infections by *Dirofilaria repens* are found more often in the southern regions of the country (Rostov region, Volgograd region, Stavropolskiy kray, Northern Caucasus, and other territories) [2, 22].

In recent years, both animal and human dirofilariosis has spread toward the North of Russia. These zoonotic nematodes are reported more frequently in the Moscow, Tyumen, and Novosibirsk regions, and in the Primorskiy and Khabarovskiy krays, where the environment favors their natural cycles [2, 22, 25]. In the last five years, dog and human dirofilariosis has been documented in 28 Russian regions [5, 21, 22, 25]. In these regions, these parasites are transmitted not only in rural areas, but also in towns, where transmission can occur the whole year round if infected dogs are present [22, 25, 29]. The key vectors of *Dirofilaria* spp. in Russia are mosquitos belonging to the *Aedes*, *Anopheles*, and *Culex* genera [2].

In Western Europe, *D. immitis* is widespread causing severe cardiac signs in dogs [20, 23]. In humans, pulmonary localizations of this parasite are observed [24], but the focal lesion in the lungs is often initially misdiagnosed as a tumor [23]. There are limited observations of other localizations of this parasite in humans, such as the chest, usually discovered in the course of coronary angiography [19], spermatic cord [17], scrotum [1, 6, 10, 15, 26], oral area [3, 9], ovaries [16], liver [11], or subcutaneous tissue [4, 7].

In this report, we present a case of dirofilariosis in a child.

## Case study

A 14-month-old child residing in a rural settlement of the Solnechnogorskiy district (Moscow region), about 56° North, was brought by the parents to a local health care center on March 2015. Significant edema at the right part of the scrotum, cyanotic tint of the affected area, and tenderness were observed. The urologist suspected torsion of the right testicle and referred the child to the surgery unit.

According to the parents, the child, who did not attend a kindergarten, was very often taken outside the home in a baby carriage, lightly dressed or even undressed in the hot days of the 2014 summer. The child was healthy before the 2014 spring but then pain and swelling developed and gradually worsened.

The child was operated in March 2015, and an inflammatory infiltrate of solid-elastic texture was removed. A very thin white filamentous worm, about 10 cm in length, was extracted from the biopsy. The worm was surrounded by necrotic tissue, hemorrhage, and it was infiltrated by eosinophils, lymphocytes, and plasma cells. The biopsy was analyzed at the Department of Medical Parasitology and Tropical Medicine of the Clinical and Diagnostic Center, Moscow State Medical University.

In April 2015, the child was in good health and no abnormal signs were observed, except minor residual edema and induration in the area of the surgical intervention. A case of dirofilariosis was suspected. No microfilariae were detected in the blood. PCR testing of a blood sample tested negative for *D. repens*.

**Figure 1.**
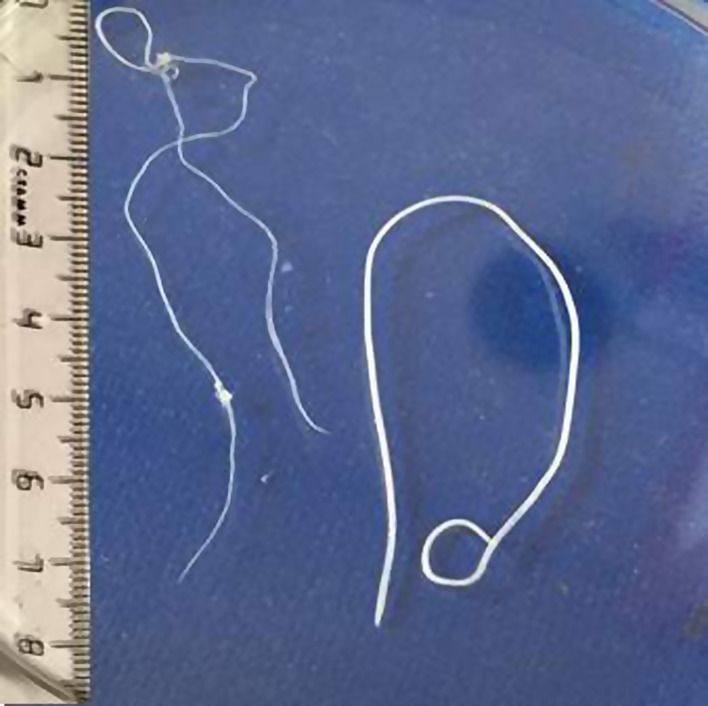
Immature female worm of *Dirofilaria immitis* (left) isolated from the scrotum of a child living near Moscow, present case, and an immature female worm of *Dirofilaria repens* (right) isolated from another patient in Russia, for comparison.

The morphological examination of the worm showed that it was an immature female (no larvae were present in the uterus) of 130 mm in length and 0.95 mm in width (Fig. 1).

The anterior and posterior ends of the body were slightly narrowed and had a roundish shape. The anus was at 0.8 mm from the tail. The cuticle was white with tender cross striations. No pectiniform longitudinal cross striations, typical for *D. repens*, were observed. Fine “cuts” located lengthwise on the cuticle were present. The uteri were formed and large nuclei were clearly visible in the notochord cells. Based on its morphology, the worm was identified as *D. immitis*.

To confirm the morphological identification of the worm, fragments from the worm isolated from the patient and from reference adult worms belonging to *D. repens* and *D. immitis* were tested by PCR using species-specific primers.

The *D. repens* specific primers were DIR-3 F-5′–CCGGTAGACCATGGCATTAT–3′, and DIR-4 R-5′–CGGTCTTGGACGTTTGGTTA–3′, amplifying a fragment of 246 base pairs. Amplification consisted of 48 cycles at 94 °C for 30 s, 50 °C for 30 s, and 72 °C for 1 min [27].

**Figure 2.**
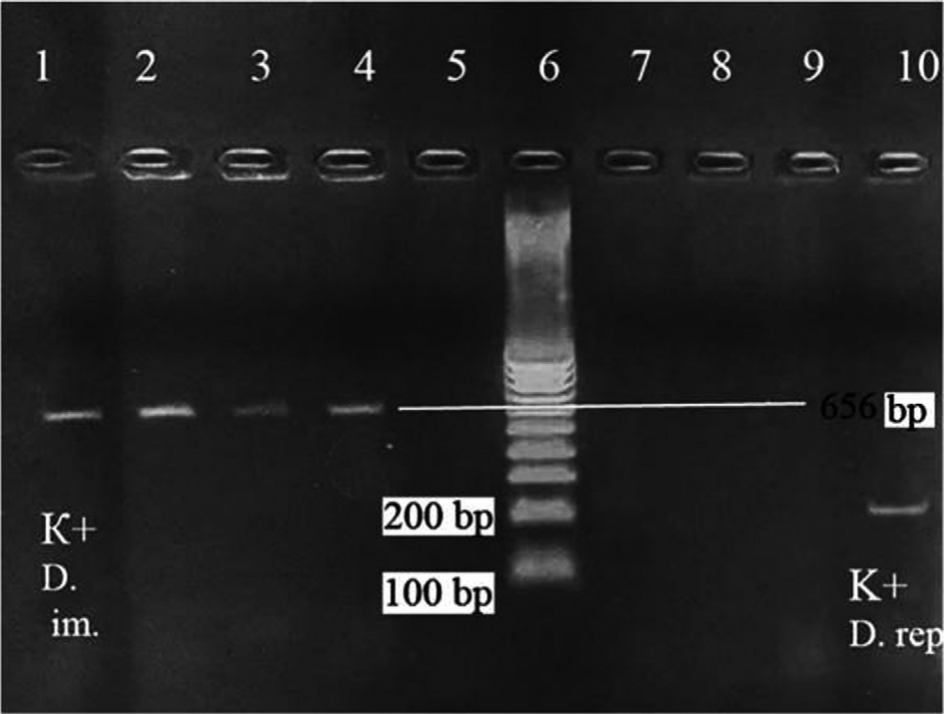
Amplicon products on agar gel electrophoresis. Lane 1, positive control of *Dirofilaria immitis*; lanes 2–4, samples from the worm removed from the patient and amplified by *D. immitis* specific primers; lane 5, negative control; lane 6, molecular mass marker; lanes 7–9, samples from the worm removed from the patient and amplified by *Dirofilaria repens* specific primers; and lane 10, *D. repens* positive control.

The *D. immitis* specific primers were CO1 int F-5′–TGATTGGTGGTTTTGGTAA–3′, and CO1 int R-5′–ATAAGTACGAGTATCAATATC–3′, amplifying a fragment of 656 base pairs. Amplification consisted of one cycle at 94 °C for 30 s, then 30 cycles at 94 °C for 1 min, 50 °C for 2 min, and 72 °C for 5 min [13]. The PCR products were visualized on a 1.5% agar gel. The worm isolated from the patient displayed the PCR pattern of *D. immitis* (amplified fragment of 656 bp), whereas no amplification was observed when the DNA was amplified by the *D. repens* specific primer pairs (Fig. 2).

## Discussion

In Russia, data on the prevalence of human dirofilariosis are limited, since the official notification and registration of clinical cases have been introduced very recently. According to Nagornyi and Krivorotova [14], *D. immitis* affects 97.2%, 29.8%, and 33% of owned and stray dogs of the Volgograd and Rostov regions, and of the Krasnodar Krai, respectively. Both *D. immitis* and *D. repens* were detected in 24.8% of dogs of the Rostov region. *D. immitis* was also documented in dogs of the Adygei Republic [14]. In the Moscow region, *D. immitis* was found in 122 dogs (3.6%) and *D. repens* in 22 dogs (0.6%) [28]. *D. immitis* was detected in 19 dogs (36.5%) and 31 dogs (29.2%) with microfilariae of the Voronezh region [30] and the Bashkiria Republic [18], respectively. Although there is very little information on the circulation of *D. immitis* among dogs in Siberia, this parasite was detected in 61 dogs (43.6%) with microfilaremia in Khabarovsk city [8].

This is the first documented report of human dirofilariosis due to *D. immitis* in the Russian Federation. The child acquired the infection at 14 months of age. No specific clinical signs and/or symptoms were observed. The parasite was identified by means of morphological examination of the worm and confirmed by molecular analysis. There is a need for more thorough and detailed analysis of each case of human dirofilariosis. We cannot exclude that *D. immitis* may be more frequent in humans in the Russian Federation than previously reported, since in most cases the worm is not identified at the species level.

In humans, *D. repens* is localized most frequently under the conjunctiva, more rarely subcutaneously in areas with areolar cellular tissue [2, 12, 21]. Over the past few years, atypical localizations of *D. repens* in humans (e.g., scrotum with signs of acute inflammation, oscheoma, spermatic cords, testicle, ovaries, penis, fallopian tube, pleura, mesentery, omentum, intestinal wall, and mucous tunic of the mouth) have become more frequent [12, 25], with the same trend as in other countries of Europe [1, 4, 7, 10, 15–17, 19, 23]. A specific sign of dirofilariosis is the sense of moving and crawling of something inside of the intumescence, tumor, or subcutaneous node [6].

Detailed assessments of epidemiological data would also be very useful and helpful in clarifying the epidemiological situation.
